# Development of a risk-stratification scoring system for predicting risk of breast cancer based on non-alcoholic fatty liver disease, non-alcoholic fatty pancreas disease, and uric acid

**DOI:** 10.1515/med-2022-0462

**Published:** 2022-03-31

**Authors:** Chuntian Hong, Yonghao Yan, Liyang Su, Debo Chen, Changqing Zhang

**Affiliations:** Department of Ultrasound, Quanzhou First Hospital Affiliated to Fujian Medical University, Quanzhou 362000, China; Department of Breast Surgery, Quanzhou First Hospital Affiliated to Fujian Medical University, Quanzhou 362000, China; Department of Gastroenterology, Quanzhou First Hospital Affiliated to Fujian Medical University, Quanzhou 362000, China

**Keywords:** breast cancer, non-alcoholic fatty liver disease, non-alcoholic fatty pancreas disease, uric acid, risk-stratification scoring system

## Abstract

Many breast cancer patients have both non-alcoholic fatty liver disease (NAFLD) and non-alcoholic fatty pancreas disease (NAFPD). Consequently, we hypothesized that NAFPD and NAFLD were associated with breast cancer, and aimed to build a novel risk-stratification scoring system based on it. In this study, a total of 961 patients with breast cancer and 1,006 non-cancer patients were recruited. The clinical characteristics were collected and analyzed using logistic analysis. Risk factors were assessed by a risk rating system. Univariate analysis showed that body mass index, triglyceride, total cholesterol, NAFLD, NAFPD, low-density lipoprotein, and uric acid (UA) were significantly related to breast cancer. Among them, NAFLD, NAFPD, and UA were independent risk factors related to breast cancer identified by multivariate analysis. The risk assessment model was established based on these factors and demonstrated that the odds ratio sharply increased with the rising scores. Compared with the low-risk group, the odds ratio in the intermediate- and high-risk groups were 1.662 (1.380–2.001) and 3.185 (2.145–4.728), respectively. In conclusion, the risk-stratification scoring system combining NAFLD, NAFPD, and UA can accurately predict the occurrence of breast cancer.

## 1 Introduction

Breast cancer is the most commonly diagnosed cancer and the leading cause of cancer death among women, with approximately 2.1 million new cases and an estimated 0.6 million deaths reported in 2018 [1]. Breast cancer patients in early stages can be cured by local and systemic treatment using surgery and chemotherapy, while the prognosis of breast cancer patients in advanced stage was poor because of recurrence or distant metastasis [2]. Many risk factors for breast cancer have been reported, including genetics, diet, lifestyle, hormonal replacement therapy, alcohol consumption, obesity, and breastfeeding [3,4,5,6,7]. Currently, the growing proportion of obesity worldwide has led to a dramatic rise in patients with metabolic syndrome and even increased risk of certain malignancies, such as breast cancer. Therefore, many studies researched the relationship between breast cancer and metabolic abnormalities [3], and reported that metabolic syndrome was associated with breast cancer [8,9,10].

Non-alcoholic fatty liver disease (NAFLD) is one of the most common chronic liver diseases, which is closely related to insulin resistance, metabolic syndrome, and abdominal obesity. In addition, more and more studies have shown that NAFLD is a multi-system disease with extrahepatic complications, such as cardiovascular disease, chronic kidney disease, pulmonary insufficiency, and extrahepatic malignancies [11,12]. Bilici et al. [13] found that hepatic steatosis was readily detected in patients of breast cancer, which might be associated with obesity. In addition, Akhondei and Gudbrandsen [14,15] reported that breast cancer patients treated with tamoxifen might produce fatty change in the liver. On the one hand, NAFLD may be an important risk factor for the incidence or treatment effect of breast cancer, while on the other hand, long-term tamoxifen therapy may increase the risk of NAFLD that may result in the patient experiencing uncertainty regarding long-term treatment [16], suggesting that there may be a pathogenesis link between NAFLD and breast cancer.

Non-alcoholic fatty pancreas disease (NAFPD) is also an important manifestation of metabolic syndrome [17]. Pancreatic steatosis was first put forward in 1933 by Oligvie, who reported the incidence of pancreatic fat storage was higher in obese individuals than in the lean ones (17% vs 9%) [18]. After this study, Van Geenen et al. [19] suggested that pancreatic steatosis preceded NAFLD and proposed that the term NAFPD should be used. Milovanovic et al. [17] reported strong correlation between NAFLD and NAFPD, and it is likely that fatty pancreas might be one of the first manifestation of metabolic syndrome. In clinical practice, we found many preoperative patients of breast cancer had both NAFLD and NAFPD. Consequently, we hypothesized that NAFPD was associated with breast cancer, which was similar to NAFLD.

Therefore, in this study, we explored the risk factors for breast cancer and the relationship between breast cancer and NAFLD/NAFPD, and developed a novel risk-stratification scoring system based on the independent risk factors to predict the incidence of breast cancer.

## 2 Material and methods

### 2.1 Study population

This retrospective study was approved by the Ethics Committee of Quanzhou First Hospital Affiliated to Fujian Medical University. Written informed consent was not applicable due to retrospective nature of the study.

A total of 961 patients with breast cancer in Quanzhou First Hospital Affiliated to Fujian Medical University from May 2017 to August 2019 were recruited. Exclusion criteria included: (1) drinking > 20 g/day; (2) patients had serious illness at the time of examination, such as cardiovascular disease, malignancies other than breast cancer, etc.; (3) patients with hepatitis A, hepatitis B, and autoimmune hepatitis; (4) history of surgery or chemotherapy. Besides, 1,006 non-breast cancer subjects were included in this study. Exclusion criteria included: (1) drinking > 20 g/day; (2) patients with hepatitis A, hepatitis B, and autoimmune hepatitis; (3) patients with pancreatitis and chronic kidney disease; (4) patients with metabolic disorders caused by drug treatment in the past year. All patients with breast cancer in the study were preoperatively diagnosed by upper endoscopy and preoperatively staged with barium radiography, computed tomography (CT), or endoscopic ultrasonography. All non-breast cancer subjects were confirmed non-breast cancer by the breast ultrasound examination, and some also underwent mammography. Hepatitis A was diagnosed by detection of immunoglobulin M (IgM) antibodies against HAV using colloidal gold strip [20]. Hepatitis B was diagnosed by detection of anti-HBc, anti-HBs, and anti-HBe antibodies using Enzyme-linked immunosorbent assay (ELISA) [21]. Autoimmune hepatitis was diagnosed by detection of serum aspartate transaminase, alanine transaminase, γ- glutamyl transferase, and γ-globulins (mainly IgG) [22]. Demographics of all participants were recorded, including age, height, weight, systolic blood pressure, and diastolic blood pressure.

### 2.2 Laboratory inspection

After fasting overnight (fasting for more than 8 h), all subjects underwent laboratory tests measured by automatic biochemical analyzer (DXC800, Beckman Coulter, USA), consisting of triglyceride (TG), total cholesterol (TC), high-density lipoprotein (HDL), low-density lipoprotein (LDL), uric acid (UA), alanine aminotransferase (ALT), and glutamic oxaloacetic transaminase (GOT).

### 2.3 Diagnosis of NAFLD and NAFPD

All subjects were at least 12 h on an empty stomach and in supine position during examination. The ultrasound examinations were performed by a qualified and experienced radiologist using a high-resolution ultrasound machine equipped with a 5 MHz convex-array probe (Philips iU Elite; Bothell, Washington). The data were evaluated by another experienced radiologist to ensure unbiased evaluation.

NAFLD was diagnosed as the presence of at least two of the following findings (excluding excessive alcohol consumption and viral or autoimmune liver disease): diffusely increased echogenitic (“bright”) liver with liver echogenicity greater than kidney or spleen, vascular blurring, and deep attenuation of ultrasound signal [23].

The diagnosis of NAFPD was based on the previous literature criteria [24]: Pancreatic echogenicity was compared to the liver echogenicity at the same depth on a longitudinal scan taken near the abdominal midline, or compared to the echogenicity of renal cortex if the liver also showed increased echogenicity. NAFPD was diagnosed if an increased echogenicity of pancreatic body over the kidney or liver echogenicity was observed during ultrasonography.

### 2.4 Statistical analysis

Data were analyzed by SPSS version 18.0 (SPSS Inc, Chicago, IL, USA). Continuous data were expressed as mean values ± SD and analyzed by unpaired two-tailed Student’s *t*-test. Categorical variables were presented as counts (percentages) and analyzed with *χ* ^2^ test. Univariate and multivariate logistic regression analysis were performed to assess the risk factors of breast cancer, and the odds ratio (OR) and 95% confidence interval (95% CI) were calculated. Variables with a value of *P* < 0.05 in the univariate analysis were subsequently included in a multivariate logistic regression analysis. The risk scoring system was used to evaluate the associations between risk predictors and breast cancer incidence. *P* values <0.05 were considered statistically significant.


**Ethics approval:** This retrospective study was approved by the Ethics Committee of Quanzhou First Hospital Affiliated to Fujian Medical University. All procedures performed in studies involving human participants were in accordance with the ethical standards of the institutional and/or national research committee and with the 1964 Helsinki declaration and its later amendments or comparable ethical standards.

## 3 Results

### 3.1 Demographic and clinical characteristics

The demographic and clinical characteristics of subjects are summarized in Table 1. There were significant differences in body mass index (BMI), systolic blood pressure, ALT, TC, UA, NAFLD history, and NAFPD history between breast cancer and non-breast cancer groups (*P* < 0.05), while no significant differences were observed between the two groups in terms of age, diastolic blood pressure, GOT, TG, HDL, and LDL.

**Table 1:** General characteristics of patients with or without breast cancer

Characteristics	Breast cancer (*N* = 961)	No breast cancer (*N* = 1,006)	*P*
Age (years)	50.0 ± 10.9	50.6 ± 10.9	0.220
BMI (kg/m^2^)	23.7 ± 2.9	22.7 ± 2.3	<0.001
Systolic blood pressure (mmHg)	130.7 ± 18.5	127.2 ± 15.3	<0.001
Diastolic blood pressure (mmHg)	81.4 ± 10.6	79.4 ± 9.5	0.146
ALT (U/L)	21.0 ± 14.8	22.4 ± 6.7	0.004
GOT(U/L)	23.3 ± 11.6	22.8 ± 5.1	0.315
TG (mmol/L)	1.33 ± 0.86	1.26 ± 0.70	0.074
TC (mmol/L)	5.45 ± 1.06	5.32 ± 0.98	0.007
HDL (mmol/L)	1.40 ± 0.31	1.41 ± 0.32	0.609
LDL (mmol/L)	3.48 ± 0.90	3.33 ± 0.82	0.248
UA (mmol/L)	306.5 ± 78.3	295.8 ± 61.2	0.001
History of NAFLD, *n* (%)	255 (26.5)	172 (17.1)	<0.001
History of NAFPD, *n* (%)	572 (59.5)	467 (46.4)	<0.001

Abbreviations: BMI, body mass index; ALT, alanine aminotransferase; GOT, glutamic oxaloacetic transaminase; TG, triglyceride; TC, total cholesterol; HDL, high-density lipoprotein; LDL, low-density lipoprotein; UA, uric acid; NAFLD, non-alcoholic fatty liver disease; NAFPD, non-alcoholic fatty pancreas disease.

### 3.2 Risk factors associated with breast cancer

Since NAFLD could lead to abnormalities in liver function indicators, such as ALT and GOT, these two indicators were excluded from the logistic analysis to prevent repeated effects of NAFLD. Then, univariate analysis showed that BMI, TG, TC, LDL, UA, NAFLD, and NAFPD were significantly related to breast cancer (*P* < 0.05), while there were no significant association between breast cancer and the clinical characteristics including age, systolic blood pressure, diastolic blood pressure, and HDL (*P* > 0.05; Table 2). After removing mixed factors, multivariate analysis further revealed that NAFLD, NAFPD, and UA were independent risk factors related to breast cancer (*P* < 0.05; Table 3).

**Table 2:** Univariate analysis of factors associated with breast cancer

Characteristics	Odds ratio (95% CI)	*P*
Age		0.720
<65	Reference	
≥65	0.947 (0.701–1.278)	
BMI		0.002
<28	Reference	
≥28	1.710 (1.208–2.420)	
Systolic blood pressure		0.066
<140	Reference	
≥140	1.207 (0.987–1.475)	
Diastolic blood pressure		0.862
<90	Reference	
≥90	0.979 (0.769–1.246)	
TG		
Normal (<1.17 mmol/L)	Reference	
Low abnormal (1.17–2.25 mmol/L)	1.389 (1.018–1.896)	0.038
High abnormal (>2.25 mmol/L)	1.582 (1.069–2.341)	0.022
TC		
Normal (<5.18 mmol/L)	Reference	
Low abnormal (5.18–6.19 mmol/L)	1.428 (1.126–1.811)	0.003
High abnormal (>6.19 mmol/L)	1.340 (1.046–1.717)	0.021
HDL		0.867
Normal (≥1.04 mmol/L)	Reference	
Abnormal (<1.04 mmol/L)	0.985 (0.825–1.176)	
LDL		
Normal (<3.37 mmol/L)	Reference	
Low abnormal (3.37–4.12 mmol/L)	1.463 (1.163–1.839)	0.001
High abnormal (>4.12 mmol/L)	1.082 (0.834–1.402)	0.554
UA		<0.001
Normal (≤420 µmol/L)	Reference	
Abnormal (>420 µmol/L)	2.778 (1.815–4.254)	
NAFLD		<0.001
No	Reference	
Yes	1.751 (1.409–2.178)	
NAFPD		<0.001
No	Reference	
Yes	1.697 (1.419–2.029)	

Abbreviations: BMI, body mass index; TG, triglyceride; TC, total cholesterol; HDL, high-density lipoprotein; LDL, low-density lipoprotein; UA, uric acid; NAFLD, non-alcoholic fatty liver disease; NAFPD, non-alcoholic fatty pancreas disease.

**Table 3:** Multivariate analysis of factors associated with breast cancer

Characteristics	Odds ratio (95% CI)	*P*	Score
BMI		0.212	
Normal	Reference		
Abnormal	1.264 (0.875–1.826)		
TG			
Normal	Reference		
Low abnormal	0.915 (0.648–1.291)	0.616	
High abnormal	1.438 (0.959–2.157)	0.079	
TC			
Normal	Reference		
Low abnormal	0.882 (0.555–1.400)	0.593	
High abnormal	1.128 (0.772–1.649)	0.533	
LDL			
Normal	Reference		
Low abnormal	1.267 (0.815–1.972)	0.293	
High abnormal	0.935 (0.641–1.363)	0.727	
UA		<0.001	
Normal	Reference		0
Abnormal	2.228 (1.435–3.459)		1
NAFLD		0.022	
No	Reference		0
Yes	1.369 (1.047–1.791)		1
NAFPD		0.001	
No	Reference		0
Yes	1.445 (1.163–1.795)		1

### 3.3 Risk-stratification scoring system in assessment on incidence of breast cancer

To estimate the risk of breast cancer occurrence, a scoring system was constructed based on the above independent predictors comprising NAFLD (no, 0 point; yes, 1 point), NAFPD (no, 0 point; yes, 1 point), and UA (normal, 0 point; abnormal, 1 point) (Table 3). In accordance with this scoring system, the patients were divided into low-risk (without a single independent predictor), intermediate-risk (1–2 independent predictors), and high-risk (3 independent predictors) groups. Compared with the low-risk group, the OR values in intermediate- and high-risk groups were sharply increased, indicating the extremely higher risk of breast cancer incidence (Table 4).

**Table 4:** Risk-stratification scoring system of breast cancer

Characteristics	Score	*N* (%)	Odds ratio (95% CI)	*P*
Low risk	0	824 (41.9)	Reference	
Intermediate risk	1–2	1,013 (51.4)	1.662 (1.380–2.001)	<0.001
High risk	3	130 (6.7)	3.185 (2.145–4.728)	<0.001

## 4 Discussion

Early diagnosis and accurate treatment are essential for breast cancer. Currently, several predictive risk models of breast cancer were established, but none of the research included NAFPD and NAFLD [25,26]. To the best of our knowledge, this was the first study to develop a risk-stratification scoring system based on NAFPD and NAFLD to predict the occurrence of breast cancer.

NAFPD is a phenotype of pancreatic steatosis, defined as the pancreatic fatty accumulation associated with obesity and metabolic syndrome [27,28], and is closely related to insulin resistance, obesity, NAFLD, type 2 diabetes, and metabolic syndrome [29]. Obesity and metabolic syndrome have been recognized as risk factors for NAFPD. Studies have demonstrated that chronic exposure of β-cells to hyperglycemia and high free fatty acids can promote increased intracellular triglyceride accumulation, resulting in decreased insulin secretion, insulin resistance, and subsequent fat replacement, thereby contributing to the development of metabolic syndrome and pancreatic fat accumulation [27,29]. Furthermore, the pancreatic fat accumulation may trigger β-cell degeneration and further accumulation of pancreatic fat, creating a vicious cycle [27]. Similar to NAFPD, increased breast cancer risk has also been reported to be associated with metabolic syndrome and its components, including obesity, diabetes, hypertension, and dyslipidemia [30]. Metabolic syndrome-induced metabolic abnormalities in breast cancer patients not only increase disease risk and tumor progression but also lead to adverse treatment responses and more treatment side effects [30]. In addition, obesity induced elevation of estrogen levels in the body is thought to be one of the mechanisms associated with breast cancer [30]. Adipose tissue is the main source of estrogen, and NAFPD is a marker of localized fat accumulation [27]. Therefore, we suspected that NAFPD might be related to breast cancer. The result in this study showed that the proportion of NAFPD in the breast cancer group was 59.5%, which was significantly higher than 46.4% in the group of no breast cancer (*P* < 0.001). Besides, the univariate and multivariate analysis further revealed that NAFPD was an independent risk factor related to breast cancer.

In recent years, the relationship between NAFLD and breast cancer has become a research hotspot. some studies reported that breast cancer was a common extrahepatic complication of NAFLD [31]. At the same time, studies also showed that endocrine therapy for breast cancer could increase the risk of NAFLD [32], suggesting that breast cancer may be related to the occurrence and development of NAFLD. The results of a case-control study by Nseir et al. [3] showed that NAFLD was associated with breast cancer, while the sample size (73 cases) included in this study was small. Another cohort study also showed an association between NAFLD and the incidence of breast cancer [33]. However, the focus of this study is on the incidence of general cancer (including breast cancer). Therefore, conventional risk factors of breast cancer such as menstrual status and age at menarche was not adjusted in the model, which might affect the accuracy of the conclusion. In this study, we analyzed the clinical data of 961 breast cancer patients and 1,006 non-breast cancer patients. The results were consistent with the abovementioned studies, confirming that NAFLD could be regarded as an independent risk factor for breast cancer.

In addition to NAFLD and NAFPD, we also found that UA was an independent risk factor for breast cancer, which was consistent with other study [34]. Yue et al. [34] suggested that high UA concentration could predict poor survival in breast cancer patients, and might serve as a potential marker for appropriate management of breast cancer patients. Therefore, in this study, we constructed a risk-stratification scoring system incorporating three predictors including NAFLD, NAFPD, and UA, by the method described by Sullivan et al. [35]. According to the scoring system, risk for breast cancer was categorized as low (0 point), moderate (1–2 points), or high (3 points). Compared with the low-risk group, the OR in intermediate- and high-risk groups were 1.662 (1.380–2.001) and 3.185 (2.145–4.728), respectively, which were significant, indicating that the scoring system could predict breast cancer risk well. Besides, the diagnosis of NAFLD or NAFPD, and the UA examination were noninvasive and convenient, which provides an easy tool for clinicians to evaluate the risk of breast cancer.

This study had some limitations which should be considered. First, this study was a retrospective analysis, which still need more data for validation. Second, this was a single-center study, and whether the models are applicable to other patient sets need further external validation. Besides, we did not evaluate known risk factors in this study such as family history of breast cancer, diabetes, breast feeding, smoking, hormone replacement therapy, and history of benign breast disease (such as atypical hyperplasia). Then, since biopsy is not clinical recommended for routine examination (except for pathological diagnosis), imaging techniques were used to diagnose NAFLD in this study, although liver biopsy is usually the reference standard for NAFLD diagnosis and staging. Finally, we analyzed only the preoperative clinicopathological data of the patients. In the future research, we should include postoperative data for analysis to better reflect the practical value of this model.

## 5 Conclusion

NAFLD, NAFPD, and UA were independent risk factors related to breast cancer. The risk-stratification scoring system combining NAFLD, NAFPD, and UA could accurately predict the incidence of breast cancer, which may help clinicians make clinical decisions.
